# Comparing Savanna with Kiko and Spanish service sires for meat goat kid and doe performance in a humid subtropical production system

**DOI:** 10.1093/tas/txag067

**Published:** 2026-05-17

**Authors:** Lauren K Stevens, Emily G Hayes, Richard Browning

**Affiliations:** Department of Food and Animal Sciences, Tennessee State University, Nashville, TN 37209-1561, United States; Department of Food and Animal Sciences, Tennessee State University, Nashville, TN 37209-1561, United States; Department of Food and Animal Sciences, Tennessee State University, Nashville, TN 37209-1561, United States

**Keywords:** breed, crossbreeding, fertility, meat goat, Savanna, weaning weight

## Abstract

Savanna meat goats were introduced to the United States from South Africa in the mid-1990s. To date, the breed has not been evaluated for relative performance attributes in the U.S. or elsewhere on a considerable scale. In this study, Savanna sires were evaluated for kid preweaning traits and doe herd reproductive traits over six years in two separate but concurrent trials. In one trial, Kiko base does were bred to Savanna (*n* = 17) and Kiko (*n* = 13) sires, with 547 doe exposures producing 628 kids. In another trial, Spanish base does were bred to Savanna (*n* = 17) and Spanish (*n* = 13) sires, with 442 doe exposures producing 457 kids. Savanna sires produced heavier (*P* < 0.01) kids at birth than Kiko and Spanish sires. Savanna-sired kids did not differ significantly from Kiko- or Spanish-sired kids for mean weaning weight or preweaning kid survival rate. Service sire breed did not affect doe kidding rate or litter size at parturition in either trial. Savanna service sires produced heavier mean litter weight at birth (*P* < 0.01) compared to Kiko and Spanish sires. Service sire breed did not affect litter weight at weaning in either trial. Litter size at weaning per doe exposed was smaller (*P* = 0.05) for Savanna service sires compared to Spanish service sires. In summary, crossbreeding with Savanna sires increased kid and litter weights at birth, but did not modify kid or litter weights at weaning compared to straightbreeding with Kiko or Spanish sires. Sire breed did not affect doe kidding rate or preweaning kid survival rate.

## Teaser

Savanna sires were assessed for possibly enhancing meat goat performance as an alternative breed choice compared with Kiko and Spanish sires in a humid subtropical production system.

## Introduction

Meat goat production is a nontraditional livestock industry in the United States. For producers, the popularity of these animals is due to an increased demand for goat meat by an increasingly diverse U.S. consumer base and their sustainability in a small-scale livestock management system. According to USDA census data, the meat goat inventory increased from under 0.5 million in 1987 to approximately 2 million in 2025 (USDC 1989; USDA 2025). The domestic U.S. meat goat harvest produced an estimated 19,632 MT in 2023 (USDA 2024). The Food and Agriculture Organization (FAO) reported an increase in metric tons of goat meat imported from 142 MT in 1987 to 10,300 MT in 2023 (FAOSTAT 2025). The U.S. exported 127 MT in 2023, establishing the U.S. as a net importer of goat meat. The U.S. was the second largest export destination of goat meat on the global market in 2023.

Over 500 goat breed populations in the world are listed by the FAO and only a small portion of those have been evaluated for capacity to meet consumer demand (Shrestha and Fahmy 2005). Scientific knowledge of meat goat breeds is limited, and most goat producers often do not have ample resources to consider when selecting breeds. Improving genetic management through better breed characterization, selection, and utilization could increase domestic production in the U.S. and elsewhere.

The South African Boer goat was imported into the United States in the 1990s. It quickly became the predominant meat goat breed because of their large body size and muscular conformation. Although widely used to improve meat goat productivity, subsequent research and producer experiences revealed limitations of the Boer compared to other breed alternatives for various fitness traits (Browning et al. 2011; Pellerin and Browning 2012; Wang et al. 2017). The Savanna meat goat breed is another import from South Africa. The breed has been referred to as the “white Boer goat” and is recognized as one of the three primary meat goat breeds developed in South Africa (Campbell 2003; Pieters et al. 2009; Casey and Webb 2010). The Boer and Savanna goat breeds are defined as different breeds, but the two have similar genetic compositions (Visser et al. 2004; Pieters et al. 2009; Mdladla et al. 2016); the latter is possibly a descendant of the former (Mohlatlole et al. 2015). The two breeds may be considered of the same biotype, but the Savanna has been characterized as a smaller, but hardier breed (Casey and Webb 2010; Visser 2018). The description of being a hardier breed increased interest among U.S. producers in using Savanna as a viable alternative to Boer, but the relative hardiness of Savanna has not been documented through controlled studies. As a distinct breed, the Savanna breed needs to be analyzed for numerous breed qualities and its potential in meat goat production systems relative to other breed options. The objective of this study was to evaluate the Savanna meat goat as a service sire breed compared with Kiko and Spanish sire breeds for birth to weaning progeny performance and the reproductive performance of mated does. It was hypothesized that service sire breeds would differ for preweaning kid and litter weight traits, but not for doe reproductive traits.

## Materials and methods

### Herd management and data collection

Across six production years (2013–2018), the herd for this study was semi-intensively managed and data were collected from dams and kids located on the research station at the Tennessee State University Research Farm (36°10′ N, 86°49′ W). The farm is located along the Cumberland River in Nashville. This area has a humid subtropical climate and receives approximately 1,222 mm of rain throughout the year. Savanna (*n* = 17), Kiko (*n* = 13), and Spanish (*n* = 13) sires were used on the study. Across 11 contemporary groups, 1085 kid records and 989 doe exposure records were included in the study evaluation (Table 1). Mature service sires were selected randomly from multiple source herds in several states within each breed without the use of breeding values. The Kiko and Savanna sires were pedigreed and represented various sire lines. The 17 Savanna sires were from 9 states across the country and 10 breeder herds with only one pair of siblings represented within the sire group. There was no Spanish Goat registry that maintained pedigrees in the U.S. at the time of this study, so genetic diversity within the Spanish sire group was based on using sires representing various ranch-labeled genetic lines identified in a targeted breed conservation effort (Sponenberg et al. 2019). Study goats were managed in accordance with the practices outlined in the Guide for the Care and Use of Agricultural Animals in Agricultural Research and Teaching (FASS 2010) as approved by the TSU Institutional Animal Care and Use Committee.

**Table 1:** Number of doe exposures and kid records by service sire breed within doe breeding trials.

	Doe trial			
	Kiko-influenced does		Spanish-influenced does	
Trait	Kiko sires	Savanna sires	Spanish sires	Savanna sires
**Does mated, n**	282	265	221	221
**Kids born, n**	325	303	240	217
**Kids weaned, n**	225	198	186	160

The herd grazed primarily cool-season tall fescue (*Festuca arundinacea*) and warm-season Bermudagrass (*Cynodon dactylon*). Other grasses, legumes, and broadleaf weed and woody plant species were available in the pastures for consumption. Pasture forage intake was supplemented with orchardgrass hay (*Dactylis glomerata*) for ad libitum consumption. Animals also had free access to water and a goat-specific mineral supplement. During the 6-week fall breeding period, a pelleted, unmedicated goat grower supplement (16% CP, 3.04 Mcal/kg of DE, as- fed) was fed at 454 g/d. During the winter and spring (December-May), does were fed whole cottonseed at a rate of approximately 454 g/d per animal. During the 2014, 2015, and 2016 kidding seasons, kids took part in a creep-feeding study (Hayes et al. 2019). Kids were split into creep and non-creep feeding groups at 30-d of age, balanced by sire breed, litter type, and sex. The kids in the creep group were offered a pelleted, unmedicated grower supplement (16% CP, 3.04 Mcal/kg of DE, as- fed) for ad libitum consumption from 30-d of age to weaning. Daily consumption of creep feed averaged 71 g per kid . During the creep-feeding periods, dams were not fed whole cottonseed or pelleted supplement.

Does were bred once a year in either October or December. Does were bred in single-sire mating pens using a pair of two-breed mating trials. One trial consisted of Savanna and Spanish sires that were bred to Spanish-influenced base does (straightbred Spanish and reciprocal Boer x Spanish F_1_ does). The other trial consisted of Savanna and Kiko sires that were bred to Kiko-influenced base does (straightbred Kiko and reciprocal Boer x Kiko F_1_ does). Many of the does used on this study and kids evaluated were produced as part of prior projects at this location (Wang et al. 2017; Hayes et al. 2019; Khanal et al. 2019). In the last four years of the study, Savanna-sired replacement doelings and their Kiko- and Spanish-sired cohorts born on study entered the breeding herd within their respective trials. Kiko-sired and Savanna x Kiko-base replacement doelings entered the Kiko breeding trial. Spanish-sired and Savanna x Spanish-base replacement doelings entered the Spanish breeding trial. Within each trial, half of the does were assigned to Savanna sires and the other half assigned to the base sire breed. Sire assignments for each doe alternated annually within the trials. Trials and service sire breed within trial were balanced across the October and December months of breeding. Does were bred starting at 2 years of age. Does on study ranged from 2 to 10 years of age. Doe age was balanced across service sire breeds. Younger does were bred as one cohort and older does as one cohort, such that doe age was not balanced across breeding months in most years. Bucks were fitted with marking harnesses to assist in estrus detection. Spanish and Kiko service sires were assigned to approximately 10 does to one buck. Savanna service sires were assigned approximately 20 does per buck because each Savanna sire was bred to does in both trials. Outside of the breeding period, does of both trials were managed together as one herd.

The kidding season took place from late February to early May. Does were vaccinated a month before the start of kidding season against pneumonia and clostridial diseases. Does delivered kids on pasture with access to shelter. All does received anthelmintic treatment at parturition. During the year, does or kids that exhibited signs of internal parasitism (eg submandibular edema, lethargy, loss of body condition, diarrhea, anemia) were treated with anthelmintics. At birth, kids were ear tagged for identification and an iodine dip was applied to the navel. Kid body weights were recorded each production year at time of birth and weaning. Kids were weighed within 12–24 hours of birth. Kids were weaned at a group mean age of 90 days. Average daily gain was then calculated from these recorded weights. Kid survival was documented from time of kidding to time of weaning. Kids that survived were coded “1” and those that did not were coded “0.”

Doe reproductive traits included parturition rate, litter weight at birth and weaning, and litter size at birth and weaning. Litter size was calculated by recording the number of live births and the number of weaned kids for every doe exposed during breeding. Adopted kids were classified as “fosters” and considered part of the litter for the foster dam when weaning traits were recorded. Failure of a doe to birth or wean a kid was recorded as “0.” Hand-raised kids were not credited to the birth dam for weaning traits. Doe reproductive traits were evaluated based on three doe populations: (1) does exposed to bucks [ie whole-herd], (2) does that kidded, and (3) does that weaned kids.

### Data analysis

Statistical testing of data was performed using SAS modeling procedures (SAS 2011) For statistical analysis of birth to weaning kid data, the birth weight, average daily gain, and weaning weight data were analyzed using MIXED model analysis. Each trial was tested separately. Survival rate data were analyzed using GLIMMIX model using a binomial distribution. Sire breed, kid sex, age of dam, litter type, and two-way interactions of sire breed with the latter three main effects were considered as independent variables in the full model for the evaluation of preweaning kid traits with kid age included as a covariate for ADG and weaning weight. Birth weight, average daily gain, weaning weight, and survivability were response variables. A creep feed model term was initially included as a fixed effect for kid traits but was never significant, so the term was eliminated from further consideration. Random effects included day of weaning contemporary group (*n* = 11), which accounted for month and year of birth and individual sire nested within the sire breed. Does that were 8 years old or older were grouped as single class of older does (8+) to balance the age of dam classes. Litter type at birth was modeled for birth weight and kid survival rate. Litter type at weaning was modeled for kid ADG and weaning weight. To balance the litter type classes, kids born in 13 triplet litters and 1 quadruplet litter were classified with twins as a combined multiple-kid litter type for analysis because the represented only 4% of the total kid population. In the Kiko trial, Kiko bucks sired 1 quadruplet and 3 triplet litters while the Savanna bucks sired 5 triplet litters. None of these litters were intact by weaning. In the Spanish trial, Spanish bucks sired 3 triplet litters while the Savanna bucks sired 2 triplet litters. By weaning, only one of the Spanish-sired triplet litters was intact by weaning.

For statistical analysis of doe reproductive performance, the litter weight data were analyzed using MIXED model analysis. Kidding rate was analyzed using GLIMMIX model using binomial distribution. Litter size data were analyzed using GLIMMIX model using Poisson distribution. For comparison of doe reproduction traits, litter weight at birth, litter size at birth, litter weight at weaning, litter size at weaning, and reproduction rates were analyzed as response variables. Service sire breed, age of doe and the interaction of service sire breed with age of doe were evaluated as independent fixed effects. Random effects included weaning contemporary grouping and individual service sire nested within the sire breed. Reproductive traits were tested within three doe populations: (1) the breeding population which was the whole-herd inclusion of all does exposed to the bucks, (2) the kidding population which was the subset of does that included only does producing kids, and (3) the weaning population which included only does that weaned at least one kid at the scheduled herd weaning date. The Kiko and Spanish doe trials were tested separately for the response variables and population subsets.

Interactions and main effects in the models were interpreted as significant sources of variation at α = .05. For significant sources of variation with more than two levels, the Tukey-Kramer means separation test was used for pairwise mean comparisons with significant differences declared at α = .05. Non-significant interaction terms were removed from the full models and reduced models with only main effects were subsequently run.

## Results

### Kid traits

Two-way interactions of sire breed with litter type or kid sex for kid traits were not significant within either of the two trials (Table 2). The only significant sire breed x dam age interactions were for weaning weight and kid survival within the Spanish-Savanna doe breeding trial, however the Tukey-Kramer post-hoc test failed to identify any significant differences among pairwise comparisons for either trait. The conservative nature of the Tukey-Kramer test would explain the lack of differences found among simple pairwise contrasts for the significant interactions.

**Table 2:** Significance levels (*P*-values) of fixed effect sources of variation for birth to weaning kid traits in meat goat kids from two doe breed mating trials.

Trait	Sire breed (B)	Sex of kid (S)	Litter type (L)^†^	Dam age (A)	B × S	B × L	B × A
	————————————- Kiko-influenced doe mating trial ————————————-						
**Birth weight**	< 0.01	< 0.01	< 0.01	0.02	0.81	0.16	0.55
**Preweaning ADG**	0.72	< 0.01	< 0.01	0.10	0.46	0.56	0.28
**Weaning weight**	0.71	< 0.01	< 0.01	0.05	0.63	0.61	0.46
**Survival**	0.22	0.99	< 0.01	< 0.01	0.20	0.84	0.30
	———————————— Spanish-influenced doe mating trial ———————————-						
**Birth weight**	< 0.01	< 0.01	< 0.01	< 0.01	0.74	0.06	0.42
**Preweaning ADG**	0.62	< 0.01	< 0.01	0.03	0.71	0.73	0.07
**Weaning weight**	0.15	< 0.01	< 0.01	0.14	0.59	0.86	0.04
**Survival**	0.34	0.89	< 0.01	0.19	0.43	0.07	0.04

† Litter type born for birth weight and survival. Litter type weaned for ADG and weaning weight.

### Birth weight

All four main effects were significant sources of variation for birth weight in both breeding trials (Table 2). Mean birth weight was higher for bucklings compared to doelings (Tables 3 and 4). In the Kiko trial, mean kid birth weight from 2-year-old dams was lower than from 6-year-old dams (Table 3). In the Spanish trial, kid birth weights from 2-year-old dams were lighter than from dams 7 years of age and older (Table 4). Kids born as singles had a higher mean birth weight than for kids born in multi-kid litters (Tables 3 and 4). When the twins and triplets were separated, each type differed (*P* < 0.01) in the Kiko trial (singles = 3.32 ± 0.08 kg; twins = 2.81 ± 0.07 kg; triplets = 2.15 ± 0.12 kg) and in the Spanish trial (singles = 3.22 ± 0.08 kg; twins = 2.75 ± 0.08 kg; triplets = 2.14 ± 0.15 kg). Classifying litter type as two or three levels did not impact other model terms in the full model. Savanna sires produced kids with a higher mean birth weight compared to Kiko sires (Table 3) and Spanish sires (Table 4).

**Table 3:** Effect of sire breed and non-genetic factors on birth to weaning traits of meat goat kids born to Kiko-influenced does.

Variable	Birth weight, kg	Preweaning ADG, g	Weaning weight, kg	Survival, %
**Sire breed**				
** Kiko**	2.91 ± 0.07^a^	123.8 ± 5.3	14.9 ± 0.6	89.1 ± 4.5
** Savanna**	3.18 ± 0.07^b^	122.4 ± 5.4	15.1 ± 0.6	81.5 ± 6.7
**Litter type** ^1^				
** Single kid**	3.31 ± 0.08^a^	137.4 ± 5.1^a^	16.5 ± 0.6^a^	89.8 ± 4.0^a^
** Multiple kids**	2.77 ± 0.07^b^	108.8 ± 5.1^b^	13.4 ± 0.6^b^	80.4 ± 5.9^b^
**Sex of kid**				
** Male**	3.22 ± 0.07^a^	134.5 ± 5.1^a^	16.3 ± 0.6^a^	85.7 ± 4.8
** Female**	2.87 ± 0.07^b^	111.7 ± 5.1^b^	13.7 ± 0.6^b^	85.8 ± 4.8
**Age of dam**				
** 2**	2.84 ± 0.09^a^	112.3 ± 6.3	13.5 ± 0.6^a^	81.1 ± 7.1^ab^
** 3**	3.02 ± 0.08^ab^	120.0 ± 5.9	14.6 ± 0.6^ab^	88.5 ± 4.7^ab^
** 4**	2.98 ± 0.09^ab^	129.6 ± 6.1	15.5 ± 0.6^b^	86.8 ± 5.5^ab^
** 5**	3.05 ± 0.09^ab^	131.4 ± 6.3	15.7 ± 0.6^b^	91.0 ± 4.3^a^
** 6**	3.21 ± 0.10^b^	126.8 ± 6.8	15.4 ± 0.6^ab^	95.0 ± 2.8^a^
** 7**	3.08 ± 0.10^ab^	122.3 ± 7.5	14.9 ± 0.6^ab^	66.3 ± 11.6^b^
** 8+**	3.12 ± 0.11^ab^	119.3 ± 8.6	15.2 ± 0.6^ab^	77.5 ± 10.0^ab^

1 Litter type born for birth weight and survival. Litter type weaned for ADG and weaning weight.

ab LSMeans (±SE) within trait and doe group not sharing common superscript differ (*P* < 0.05).

**Table 4:** Effect of sire breed and non-genetic factors on birth to weaning traits of meat goat kids born to Spanish-influenced does.

Variable	Birth weight, kg	Preweaning ADG, g	Weaning weight, kg	Survival, %
**Sire breed**				
** Spanish**	2.82 ± 0.09^a^	110.8 ± 5.6	13.5 ± 0.6	80.8 ± 4.2
** Savanna**	3.11 ± 0.09^b^	112.9 ± 5.6	14.1 ± 0.6	77.0 ± 4.8
**Litter type** ^1^				
** Single kid**	3.21 ± 0.08^a^	123.8 ± 5.4^a^	15.1 ± 0.6^a^	85.6 ± 3.7^a^
** Multiple kids**	2.72 ± 0.08^b^	99.8 ± 5.5^b^	12.5 ± 0.6^b^	70.4 ± 5.1^b^
**Sex of kid**				
** Male**	3.08 ± 0.08^a^	121.9 ± 5.4^a^	14.9 ± 0.6^a^	79.2 ± 4.5
** Female**	2.85 ± 0.08^b^	101.8 ± 5.4^b^	12.7 ± 0.6^b^	78.7 ± 4.4
**Age of dam**				
** 2**	2.68 ± 0.11^a^	103.8 ± 7.4^ab^	13.4 ± 0.8	67.1 ± 8.8
** 3**	2.91 ± 0.10^ab^	102.1 ± 6.5^a^	12.8 ± 0.7	82.7 ± 5.3
** 4**	2.93 ± 0.10^ab^	120.2 ± 6.5^b^	14.5 ± 0.7	83.2 ± 5.7
** 5**	3.01 ± 0.10^ab^	112.0 ± 6.7^ab^	13.8 ± 0.7	84.7 ± 5.5
** 6**	2.95 ± 0.11^ab^	120.9 ± 7.7^ab^	14.5 ± 0.9	64.8 ± 9.4
** 7**	3.10 ± 0.11^b^	108.5 ± 7.2^ab^	13.5 ± 0.8	83.2 ± 6.0
** 8+**	3.20 ± 0.11^b^	115.5 ± 7.3^ab^	14.0 ± 0.8	81.2 ± 7.2

1 Litter type born for birth weight and survival. Litter type weaned for ADG and weaning weight.

ab LSMeans (±SE) within trait and doe group not sharing common superscript differ (*P* < 0.05).

### Preweaning kid growth rate

Sex of kid and litter type were significant sources of variation for birth-to-weaning ADG in both breeding trials (Table 2). Bucklings had a higher mean ADG compared to doelings (Tables 3 and 4). Kids weaned as singles had a higher mean ADG than for kids weaned as twins (Tables 3 and 4). Age of dam significantly affected kid ADG only in the Spanish trial, whereas breed of sire did not affect kid ADG in either trial (Table 2). The mean for kid ADG from 2-year-old Spanish dams was lower than from Spanish dams 7 years of age and older (Table 4). Progeny of Savanna sires did not differ from kids of Kiko sires (Table 3) or Spanish sires (Table 4) for preweaning ADG.

### Weaning weight

Sex of kid and litter type were significant sources of variation for weaning weight in both breeding trials (Table 2). Mean weaning weight was higher for bucklings compared to doelings (Tables 3 and 4). Kids weaned as singles had a higher mean weaning weight than kids weaned as twins (Tables 3 and 4). Age of dam significantly affected kid weaning weight only in the Kiko breeding trial, whereas breed of sire did not affect kid weaning weight in either breeding trial (Table 2). Mean kid weaning weight from 2-year-old Kiko dams were lower than from Kiko dams 4 and 5 years of age (Table 4). Progeny of Savanna sires did not differ from kids of Kiko sires (Table 3) or Spanish sires (Table 4) for mean weaning weight.

### Kid survival rate 

Litter type was the only source of variation that was important for preweaning kid survival in both breeding trials (Table 2). Kids born as singles had a higher survival rate than for kids born in multi-kid litters (Tables 3 and 4). When the twins and triplets were separated for the Spanish doe trial, survival of single kids (85.9 ± 3.8%) was higher (*P* ≤ 0.01) than for twins (72.4 ± 5.4%) and triplets (45.9 ± 16.5%) with twins and triplets not differing. In the Kiko trial, preweaning survival rate for single kids remained higher (*P* = 0.01) than for twins (90.6 ± 3.6% vs. 83.1 ± 5.2%) when triplets and quadruplet kids were excluded. Least square means could not be estimated for triplet and quadruplet kids by the models used, probably because the arithmetic mean was zero both trials. Classifying litter type as two or three levels did not impact the level of significance for other terms in the full model. Dam age was important in the Kiko doe trial (Table 2). Kid survival rate from 7-year-old Kiko dams were lower than from Kiko dams 5 and 6 years of age (Table 4). Breed of sire did not affect preweaning kid survival (Table 2) in either trial as Savanna-sired kids did not differ from kids of Kiko sires (Table 3) or Spanish sires (Table 4).

### Doe traits

The service sire breed x age of doe interaction was not significant for any of the doe traits presented in Table 5 for the Kiko or Spanish doe trials. Age of doe affected (*P* < 0.05) all doe traits except kidding rate in the Spanish trial. Within the Kiko trial, age of doe affected (*P* < 0.05) litter size at kidding for the breeding population and all four litter weight evaluations. The primary feature of the age of doe assessment was that 2-year-old does had lower (*P* ≤ 0.05) reproductive values than one or more of the older doe groups (data not shown). The only doe performance trait significantly affected by service sire breed in both trials was litter weight at birth among does kidding. The Savanna service sires produced litters that were heavier (*P* < 0.01) at birth when compared to the Kiko and Spanish service sires (Table 5). In the Spanish trial, Spanish service sires generated a higher (*P* = 0.05) litter size at weaning when the whole-herd breeding population was analyzed (Table 5).

**Table 5:** Effects of service sire breed on doe reproductive output in different doe populations.

	Doe trial			
	Kiko-influenced does		Spanish-influenced does	
Trait by population	Kiko sires	Savanna sires	Spanish sires	Savanna sires
**Kidding rate, %**				
** Breeding** ^1^	70.7 ± 5.2	69.5 ± 5.2	77.6 ± 5.0	68.2 ± 6.0
**Litter size at birth, n** ^2^				
** Breeding**	1.11 ± 0.08	1.09 ± 0.08	1.11 ± 0.11	1.01 ± 0.10
** Kidding**	1.60 ± 0.04	1.63 ± 0.04	1.43 ± 0.05	1.45 ± 0.05
**Litter weight at birth, kg**				
** Kidding**	4.59 ± 0.14^a^	5.08 ± 0.14^b^	4.06 ± 0.19^a^	4.54 ± 0.19^b^
**Litter size at weaning, n**				
** Breeding**	0.72 ± 0.08	0.68 ± 0.07	0.85 ± 0.08^a^	0.72 ± 0.07^b^
** Kidding**	1.09 ± 0.08	1.05 ± 0.08	1.10 ± 0.07	1.03 ± 0.07
** Weaning**	1.33 ± 0.04	1.37 ± 0.05	1.29 ± 0.05	1.30 ± 0.05
**Litter weight at weaning, kg**				
** Breeding**	11.4 ± 1.3	10.8 ± 1.3	12.2 ± 1.3	11.0 ± 1.3
** Kidding**	16.5 ± 1.4	15.3 ± 1.4	15.6 ± 1.3	15.4 ± 1.3
** Weaning**	20.4 ± 1.0	20.2 ± 1.0	17.9 ± 0.9	18.8 ± 1.0

1 Defined doe population.

2 n = number of kids.

ab LSMeans (±SE) within trait and doe group not sharing common superscript differ (*P* ≤ 0.05).

## Discussion

### Kid traits

Assessing litter size, dam age, and kid sex as non-genetic influences on preweaning kid traits is common in meat goat breed evaluation studies (Goonewardene et al. 1998; Browning and Leite-Browning 2011; Supakorn et al. 2011; Tessema et al. 2022). The non-genetic effects reported here were similar to those observed earlier at this research station (Browning and Leite-Browning 2011).

The higher birth weights of Savanna-sired kids compared to Kiko- and Spanish-sired kids agreed with the Boer, Kiko and Spanish sire comparisons of Browning and Leite-Browning (2011) and other studies in which Boer bucks sired crossbred kids had higher birth weights from base breed dams than base breed bucks that sired straightbred kids from the respective base breed dams (Merlos-Brito et al. 2008; Supakorn et al. 2011; Bolacali et al. 2017; Tessema et al. 2022). Birth weight as a trait commonly thought of as the beginning of the preweaning growth period, but it also represents the end of the fetal growth period. Increased birth weight in Savanna sire matings indicated that Savanna sires enhanced fetal growth compared to Kiko and Spanish sires but did not cause dystocia when characterized as parturitions requiring assistance (Speijers et al. 2010). Browning and Leite-Browning (2011) reported that Boer had a significantly positive direct additive effect on kid birth weights, while Spanish and Kiko sires had negative effects. This corresponds with the increased birth weights seen in the current study for Savanna-sired kids.

Weaning weight is an economically important trait because it is the first point at which market kid values are determined (Hayes et al. 2019). Weaning weight is also an initial trait used for assessing and selecting replacement doelings (Khanal and Browning 2019). Sire breed did not affect preweaning ADG or weaning weight in either trial. A producer report (Pinkerton 2011) indicated that growth rates were not improved when Savanna-sired kids were compared to Spanish-sired kids out of Spanish does. In agreement, Browning and Leite-Browning (2011) found that Boer-sired F_1_ kids did not differ for preweaning ADG or weaning weight compared to straightbred Kiko or Spanish kids as the weight advantages at birth were lost by weaning. In other studies, Boer sires failed to increase preweaning ADG and weaning weights when producing F_1_ kids compared with sires producing straightbred base breed kids (Goonewardene et al. 1998; Rhone 2005; Menezes et al. 2007) with the weight advantage at birth dissipating by weaning (Ayoade and Butterworth 1982; Oliveira 2006). To the contrary, others found that crossing with Boer sires increased preweaning ADG and weaning weights compared to base straightbred kids representing various local breeds (Merlos-Brito et al. 2008; Supakorn et al. 2011; Kalenga et al. 2015; Bolacali et al. 2017; Tessema et al. 2022). The ability of a new breed option to enhance weaning weight is partly dependent on characteristics of the base dam breed. Ssewannyana et al. (2004) demonstrated differing responses by dam breed as Boer-sired F1 kids were significantly heavier than straightbred kids at birth and weaning for one base dam breed, but not for a second base dam breed.

It is not clear why the advantage in fetal growth shown in the Savanna-sired kids by birth was not sustained during the preweaning period. Intermediate (30-day and 60-day) kid weights and ADG for a subset of study kids on the creep-feeding study indicated little separation among Savanna, Kiko, and Spanish sire breeds (Hayes et al. 2019). Factors such as maternal nutrition, internal parasite burden, and climatic conditions could affect kid growth in ways more impactful after birth than before birth within the study protocols. Although the non-genetic factors of sex and litter size exerted their typical influences on prenatal and postnatal kid growth under the conditions of this study, sire breed only affected the prenatal growth of meat goat kids.

Preweaning kid mortalities can be detrimental to the success of a meat goat breeding program if occurrences are excessive and persistent. Sire breed did not significantly affect kid survival rates from birth to weaning in either study doe trial. Sire breed effects on progeny survival were not found in studies involving Boer sires (Browning and Leite-Browning 2011; Kalenga et al. 2015; Bolacali et al. 2017; Tessema et al. 2022). A combination of dam breed and non-genetic factors such as litter size and season of birth had a greater impact on kid survival than breed of sire (Browning and Leite-Browning 2011).

### Doe traits

When selecting sire breeds to produce kids, it is important to understand how the service sires affect the reproductive output of the doe herd, particularly when naturally mated. Poor reproductive performance of the service sires in a natural mating program would counteract any expected benefits gained from the genetic potential of the selected sire breed to improve kid growth performance. Reproductive performance of the breeding herd is an economically important trait, thus the need to evaluate service sire effects on doe herd performance. Kidding rate, number of kids born, and total weight weaned are three important indicators of doe herd performance in a commercial meat goat operation. Service sire breed did not affect either of these traits, as was the case when Boer service sires were compared to Kiko and Spanish sires (Browning et al. 2011). Savanna service sires had higher doe:buck ratios than Kiko and Spanish sires without a decline in kidding rate. The doe:buck ratio disparity between breeds in the current study was not wide enough to expect an impact on pregnancy rate (Mellado et al. 1996, 2000).

Increased mean litter weight at birth for Savanna service sires was a direct consequence of the heavier progeny birth weights for Savanna sires. Litter weight differences between service sire breeds were not evident by weaning, when biological and economic assessments of doe herd performance would occur. Like the Savanna service sire effect, Browning et al. (2011) reported that litters sired by Boer bucks were heavier than litters produced by Kiko and Spanish sires at birth, but not at weaning. The cumulative effects of various factors during the nine months from breeding to weaning could affect litter size at weaning. The service sire breed effect on litter size at weaning within the Spanish doe breeding population may be more reflective of the Spanish sires than the Savanna sires; the Savanna sire values were consistent across the two breeding doe trials. The results of this study suggest that choice of service sire breed as studied here did not broadly affect doe performance by weaning.

## Conclusion

Savanna sires increased kid birth weights and doe litter weights at birth across dam breeds, but did not increase kid weaning weights or doe litter weights at weaning compared to Kiko sires or Spanish sires. There was no advantage observed in crossbred matings of Kiko or Spanish does with Savanna bucks as an alternative to straightbred matings to increase weaning weights under the conditions of this study. Service sire breed did not affect reproductive performance in the doe herd, exception for whole-herd litter size weaned in Spanish does favoring Spanish service sires over Savanna service sires. The relative performance of the Savanna sires closely aligned with earlier observations with Boer sires at this location. It is important to characterize Savanna goats relative to other breed options at the production level where the relative phenotypic merits of breed options are important for producers to consider when making breed selection and mating decisions. Further research would be beneficial to assess the value of the Savanna breed under different production systems and as a maternal component in meat goat herd management.
